# Development of a nomogram for prediction of central lymph node metastasis of papillary thyroid microcarcinoma

**DOI:** 10.1186/s12885-024-12004-3

**Published:** 2024-02-20

**Authors:** Pengjun Qiu, Qiaonan Guo, Kelun Pan, Jianqing Lin

**Affiliations:** Department of Breast and Thyroid Surgery, The Second Affiliated Hospital of Fujian Medical University, Quanzhou, China

**Keywords:** Papillary thyroid microcarcinoma (PTMC), Central lymph node dissection (CLND), Nomogram, Decision curve analysis (DCA)

## Abstract

**Background:**

Papillary thyroid carcinoma (PTC) is the most frequent malignant tumor in thyroid carcinoma. The aim of this study was to explore the risk factors associated with central lymph node metastasis in papillary thyroid microcarcinoma (PTMC) and establish a nomogram model that can assess the probability of central lymph node metastasis (CLNM).

**Methods:**

The clinicopathological data of 377 patients with cN0 PTMC were collected and analyzed from The Second Affiliated Hospital of Fujian Medical University from July 1^st^, 2019 to December 30^th^, 2021. All patients were examined by underwent ultrasound (US), found without metastasis to central lymph nodes, and diagnosed with PTMC through pathologic examination. All patients received thyroid lobectomy or total thyroidectomy with therapeutic or prophylactic central lymph node dissection (CLND). R software (Version 4.1.0) was employed to conduct a series of statistical analyses and establish the nomogram.

**Results:**

A total of 119 patients with PTMC had central lymph node metastases (31.56%). After that, age (*P* < 0.05), gender (*P* < 0.05), tumor size (*P* < 0.05), tumor multifocality (*P* < 0.05), and ultrasound imaging-suggested tumor boundaries (*P* < 0.05) were identified as the risk factors associated with CLNM. Subsequently, multivariate logistic regression analysis indicated that the area under the receiver operating characteristic (ROC) curve (AUC) of the training cohort was 0.703 and that of the validation cohort was 0.656, demonstrating that the prediction ability of this model is relatively good compared to existing models. The calibration curves indicated a good fit for the nomogram model. Finally, the decision curve analysis (DCA) showed that a probability threshold of 0.15–0.50 could benefit patients clinically. The probability threshold used in DCA captures the relative value the patient places on receiving treatment for the disease, if present, compared to the value of avoiding treatment if the disease is not present.

**Conclusion:**

CLNM is associated with many risk factors, including age, gender, tumor size, tumor multifocality, and ultrasound imaging-suggested tumor boundaries. The nomogram established in our study has moderate predictive ability for CLNM and can be applied to the clinical management of patients with PTMC. Our findings will provide a better preoperative assessment and treatment strategies for patients with PTMC whether to undergo central lymph node dissection.

**Supplementary Information:**

The online version contains supplementary material available at 10.1186/s12885-024-12004-3.

## Introduction

Papillary thyroid carcinoma is the most frequent malignant tumor in thyroid carcinoma. The clinic-biological behavior of PTC is relatively inert, with a 10-year survival of over 90% after standard management [1, 2]. According to the definition by World Health Organization (WHO), PTMC is defined as a papillary thyroid carcinoma less than 10 mm [3]. Increasing awareness of health examinations has prompted more people to take regular medical checkups, which has enabled PTMC to be detected at an early stage rather than by the time symptoms emerge. At the same time, the high-resolution ultrasound makes it possible to detect microscopic tumors that would otherwise be difficult to observe and makes the morphology of tumors clearer, reducing the probability of misdiagnosis and underdiagnosis. Thanks to the popularity of health examinations and the application of examination devices such as high-resolution ultrasound, more and more patients with asymptomatic thyroid carcinoma are detected in the early stage. In recent years, the incidence rate of PTC is obviously increasing worldwide. It is reported that the incidence of thyroid cancer in China also continues to grow, with an annual growth rate of about 20% [4]. Not only in China, but also in other countries the incidence of thyroid cancer is increasing year by year. A report from 2015 American Thyroid Association (ATA) Management Guidelines suggested that the incidence of thyroid cancer has increased threefold in the past 30 years [5]. In South Korea, the incidence of thyroid cancer increased by an average of 24.2% per year from 1999 to 2010 [6]. Although PTMC exhibits a slow progression with a relatively better prognosis, the lymph node metastasis occurs frequently in the early stages, especially the CLNM, which has been considered to be a risk for distant metastasis and recurrence [7].

The TNM classification of malignant tumors is a globally accepted standard for classifying the anatomical extent of the spread of malignant tumors. Most common tumors have their own TNM classification, and papillary thyroid cancer is no exception. According to the TNM system, patients with PTMC are labeled as cN1 or cN0 for lymph node positive or negative, respectively, based on the results of physical examination or imaging. Thyroidectomy combined with therapeutic lymph node dissection, including CLND, has now become a frequent initial surgical strategy for patients with clinical lymph node-positive (cN1) PTMC [8]. Despite this, for patients with no preoperatively detected cervical lymph node metastases, some experts advocate prophylactic central lymph node dissection (pCLND), while other clinicians believe it is unnecessary. Therefore, it remains a hot topic of debate whether to perform pCLND in PTMC patients without clinical lymph node-positive (cN0). According to ATA guidelines (version: 2015) and National Comprehensive Cancer Network (NCCN) guidelines (version: 2022), pCLND is not recommended as a routine surgical procedure in cN0 PTMC patients without high risk factors [5, 9]. Nevertheless, the necessity of pCLND for PTMC patients with cN0 has been positively highlighted in multiple guidelines from some different countries [10, 11]. Given that PTMC patients is frequently found central region lymph nodes involvement and pCLND is considered helpful to the risk reduction of recurrence and second operation, the Chinese Society of Clinical Oncology (CSCO) guidelines (version: 2018) recommended performing pCLND after a comprehensive assessment of the patient's surgical risks and benefits [12]. Although various studies committed to find the risk factors for CLNM, it is still lack of consensus and standard criterion to instruct clinicians on the indications for CLND. Therefore, it is significant to construct a prediction model to assess the lymph node involvement preoperatively to further realize the personalized precision treatment.

In this study, we aimed to establish a prediction model to assess the conditions of PTMC patients preoperatively and instruct clinicians to provide optimal surgical strategies for PTMC patients with cN0. Since existing guidelines and treatment protocols do not explicitly mention the necessity of CLND for PTMC patients with cN0 and there is no quantitative criterion to guide clinicians on the circumstances under which CLND is necessary, the decision-making resulting from such pros and cons judgments is subjective, which may lead to irregularities in treatment. Our study aims to develop a nomogram-based CLNM risk model for PTMC to help with the decision on the surgery choice, utilizing clinical data from our medical center and focusing on five independent factors: age, gender, multifocality, tumor boundary, and tumor size.

## Methods

### Ethics approval and informed consent

This research protocol was approved by the Ethics Committee of Second Affiliated Hospital of Fujian Medical University (2022 Ethical Review No. 481) and was conducted in accordance with the Declaration of Helsinki, and informed consent was obtained from all subjects or their legal guardians.

### General data

A total of 377 patients with PTMC enrolled in the Thyroid and Breast Surgery of Second Affiliated Hospital of Fujian Medical University from July 2019 to December 2021 were recruited in the current study. All data were extracted from the electronic medical record system, and no personally identifiable information was displayed in accordance with the principles of patient privacy protection. The completed information was provided in Supplemental Table 1. The PTMC diagnosis of all samples was confirmed through postoperative pathology.

Inclusion criteria were as follows:

- Patients with completed clinical and pathologic information records.
- Patients who underwent ultrasound examination before operation in our hospital without clinical abnormal findings in central lymph nodes by physical and ultrasound examination.
- Initial thyroidectomy, intraoperative frozen section and postoperative paraffin pathological diagnosis of PTMC, and surgical range including total CLND.
- Patients who were operated by the same medical team.

The exclusion criteria were as follows:

- Other types of thyroid carcinoma (thyroid follicular carcinoma, thyroid medullary carcinoma, and undifferentiated thyroid carcinoma et al.).
- Missing information of medical record and US reports of our hospital.
- History of head/neck surgery or radiology, history of other malignancy and metastatic thyroid cancer.
- PTMC patients with distant metastasis.

Moreover, the US reports were issued by more than two US physicians, and the ultrasonic characteristic records included position, boundary situation, microcalcification situation, the length/width ratio, and the peripheral blood supply of tumor. In addition, all specimens were examined by two or more pathologic physicians from Second Affiliated Hospital of Fujian Medical University. Pathological characteristics analysis included pathological type of tumor, size, multifocality (more than one lesion in unilateral thyroid lobe), and central lymph node metastasis.

### Surgical strategy

Surgeries were conducted by specialists from the same medical team and who perform at least 100 surgeries annually. All patients received thyroid lobectomy or total thyroidectomy with therapeutic or prophylactic CLND. The upper, lower, and external boundaries of CLND were lower edge of hyoid bone, upper edge level of the brachiocephalic artery and internal carotid artery sheath. The posterior boundary was anterior fascia, including all paratracheal, pretracheal and prelaryngeal lymph nodes and adipose tissues [12].

### Statistical analysis

We conducted this study to identify the relationship between clinical features and central lymph nodes (CLN) involvement in PTMC patients. A series of continuous statistical calculations were performed by using R software (Version 4.1.0; http://www.r-project.org). The chi-square test and Mann–Whitney U test was adopted for univariate analysis. Subsequently, the all data from our medical center was randomly divided into two cohorts for cross-validation: 70% for a training cohort and 30% for an internal testing cohort. The least absolute shrinkage and selection operator (LASSO) regression analysis is a regression analysis method that executes both variable selection and regularization to improve the accuracy of forecasting and the explainability of the statistical model. In order to reduce redundant factors and obviate model overfitting, LASSO regression analysis was employed to screen out the optimal variables as potential risk factors for this prediction model [13]. Variables of which the *p*-value < 0.05 from the LASSO regression were applied to multivariate logistic regression and a subsequent nomogram was constructed. The prediction efficiency of the novel predictive model was evaluated through the receiver operating characteristic (ROC) curve, the calibration curve, and the area under the ROC curve (AUC) which is also known as concordance index (C-index). Notably, C-index is specifically used for the quantification of the ability to discriminate between outcomes. After that, the net benefit at disparate threshold probabilities were quantified to further identify the clinic value of the model by use of the method of DCA. *P* < 0.05 was considered as statistically significant.

## Results

### Clinicopathological characteristics of PTMC patients

A total of 377 cases diagnosed with non-distance-metastatic histologically confirmed PTMC in Second Affiliated Hospital of Fujian Medical University from 2019 to 2021 were recruited in the current study. The ratio of male to female was approximately 1:3.60, which is similar to other studies on PTMC. Approximately 81% of the PTMC patients were < 55 years old. The majority of the specimens were solitary tumor. Approximately 68.44% of the patients were lymph node negative. The clinicopathological characteristics of the samples involved in are summarized in Table 1.

**Table 1:** Clinicopathological characteristics of the samples

	Characteristics	Number	Percentage (%)
Gender	Male	82	21.75
	Female	295	78.25
Age^a^		43.67 ± 11.23	
	≥ 55	71	18.38
	< 55	306	81.17
Tumor Size Multifocality		0.63 ± 0.21	
	Negative	295	78.25
	Positive	82	21.75
Location	Upper	87	23.08
	Middle	171	45.36
	Lower	110	29.18
	Isthmus	9	2.39
Boundary	Clear	171	45.36
	Unclear	206	54.64
A/T	> 1	181	48.01
	≤ 1	196	51.99
Microcalcification	Positive	213	56.50
	Negative	164	43.50
Blood Supply	Positive	118	31.30
	Negative	259	68.70
CLNM	Positive	119	31.56
	Negative	258	68.44

^a^The age was presented as mean ± standard deviation

### Selection of potential predictors

**Fig. 1:**
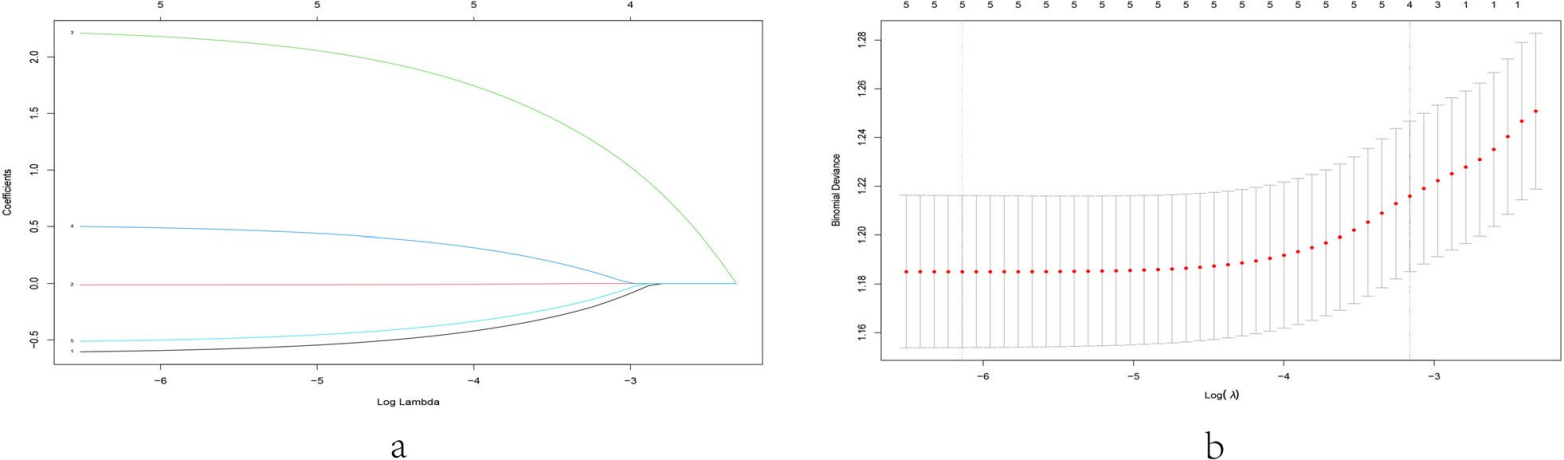
LASSO coefficient profiles of the risk factors with *P* < 0.05 (**A**). The results of the fivefold cross-validation determined the optimal value of the penalty parameter λ (λ = 0.002157022). Five independent prognostic genes for risk model construction were identified (**B**)

The univariate analysis was used to identify the relationship between the central lymph node metastasis and 9 risk factors, involving age, gender, multifocality, tumor location, tumor boundary, tumor aspect ratio, microcalcification, tumor size, and peripheral blood supply of tumors. According to the results of univariate analysis, five variables were identified to be significantly related to CLNM, including age, gender, multifocality, tumor boundary and tumor size (Table 2). Subsequently, to avoid the affection of confounding factors, the LASSO regression analysis was performed to confirm the final variables. The LASSO coefficient overview of the selected factors was showed in Fig. 1a and 5-fold cross-validation results were produced to identify the preferred value of the penalty parameter λ (λ = 0.002157022) (Fig. 1b). As a result, the finally selected potential predictors were age, gender, multifocality, tumor boundary and tumor size.

**Table 2:** Five variables were identified related to CLNM by univariate analysis

	Factors	CLNM + (119)	CLNM-(258)	X^2^/W	*P*
Gender				5.3568	0.021
	Male	35	47		
	Female	84	211		
Multifocality				4.1856	0.041
	Negative	85	210		
	Positive	34	48		
Location				Fisher^’^s	0.535
	Upper	27	60		
	Middle	58	113		
	Lower	30	80		
	Isthmus	4	5		
Boundary				4.4489	0.035
	Clear	44	127		
	Unclear	75	131		
A/T				0.13113	0.717
	> 1	55	126		
	≤ 1	64	132		
Microcalcification				0.90948	0.340
	Positive	72	141		
	Negative	47	117		
Blood Supply				0.28995	0.590
	Positive	40	78		
	Negative	79	180		
Age		42.09 ± 11.16	44.40 ± 11.22	17392	0.038
Tumor Size		0.70 ± 0.21	0.60 ± 0.22	11551	< 0.001

### Establishment and validation of the prediction model

**Fig. 2:**
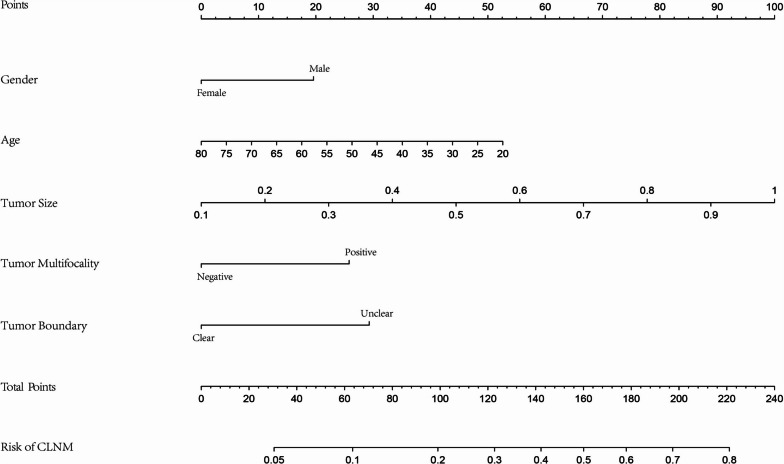
Clinical prognostic nomogram for risk of CLNM. Clinical prognostic nomogram was applied to predict the risk of CLNM by gender, age, tumor size, tumor multifocality and tumor boundary

**Fig. 3:**
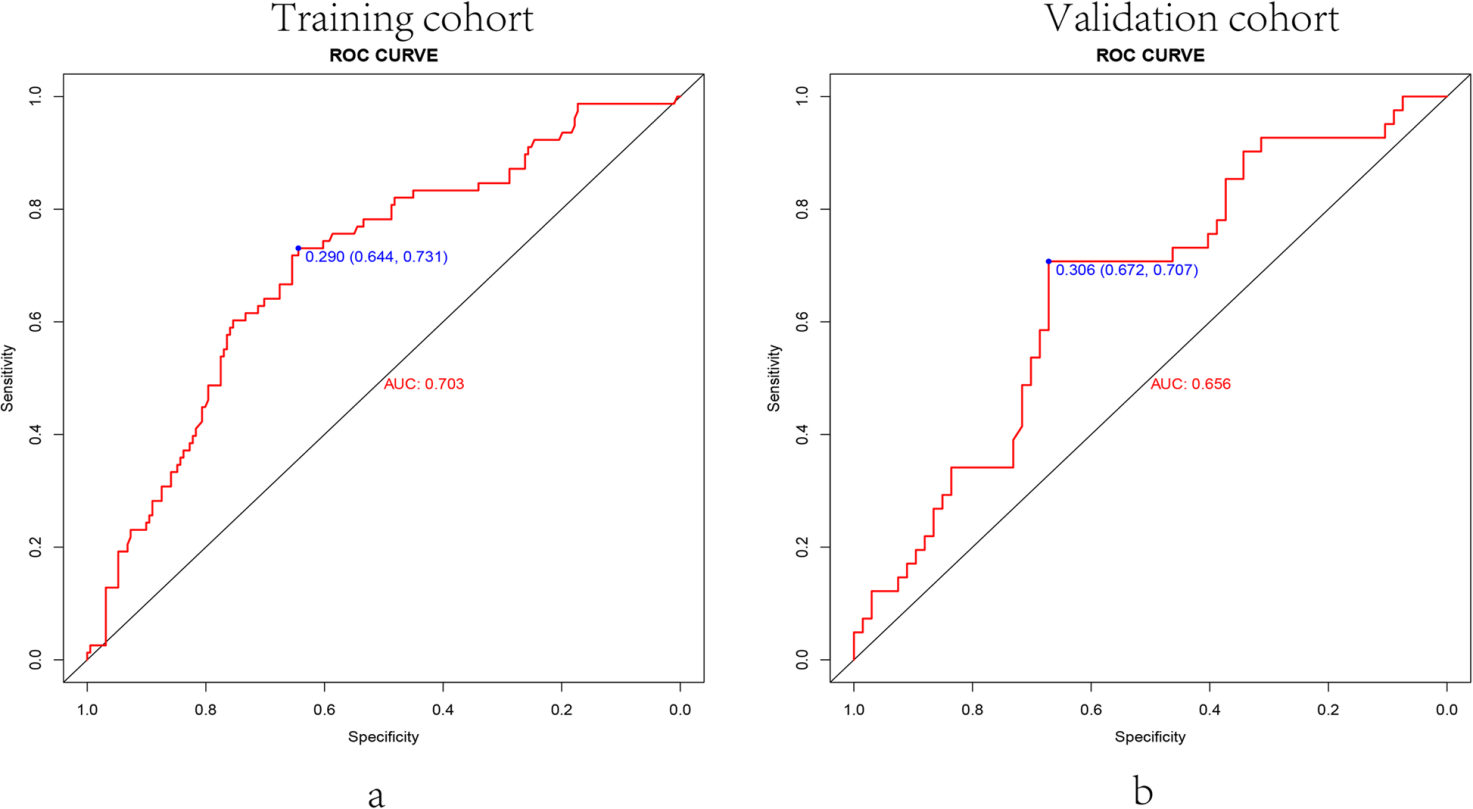
The ROC curve of nomogram in PTMC patients: The AUC of the nomogram model in the training set was 0.703 (**A**), and that in the validation set was 0.656 (**B**)

**Fig. 4:**
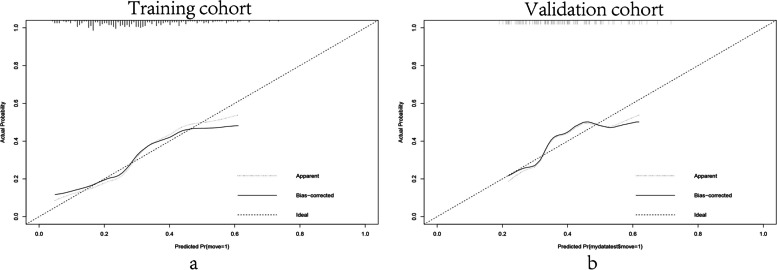
The calibration curve of nomogram in PTMC patients. The calibration curve in the training set (**A**) and validation set (**B**)

**Fig. 5:**
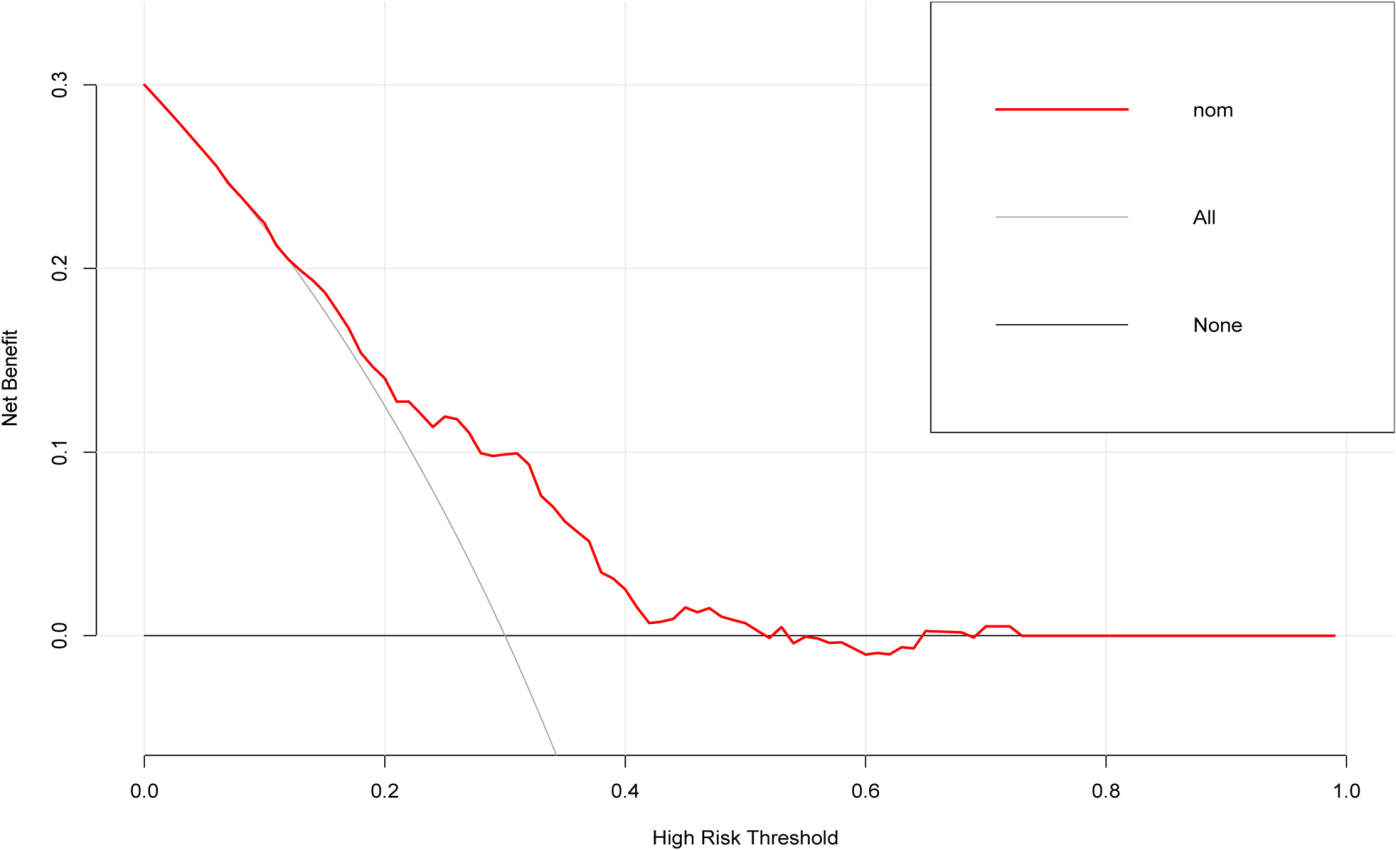
The decision curve analysis of nomogram model in PTMC patients

All data were randomly divided into two cohorts for cross-validation purposes to ensure the model's generalizability, which consisted of the training cohort (*n* = 269, 70% of all cases from our center) and the validation cohort (*n* = 108, 30% of all cases from our center). The features of 5 variables between training and validation cohorts were summarized in Table 3. To gain a more complete understanding of the relationship between the CLNM and these predictors, the multivariable logistic regression analysis was further performed and a prediction model was subsequently established. The results were visualized in the form of a nomogram, which is easier to apply in the clinical practice (Fig. 2). After that, the ROC curve and the calibration curve were performed to evaluate the prediction efficiency of the nomogram model. As shown in Fig. 3, the AUC was 0.703 for the training cohort and it was 0.656 for the validation cohort. The C-index of this model in the training cohort was 0.703 (95%CI: 0.640–0.731), and the one in validation cohort was 0.656 (95%CI: 0.672–0.707). The calibration curve of CLNM risk nomogram in PTMC indicated a good consistency in both training and validation cohorts, and all the mean absolute error < 0.05, which suggested the good fit of the predictor model (Fig. 4). The probability threshold used in DCA captures the relative value the patient places on receiving treatment for the disease, if present, compared to the value of avoiding treatment if the disease is not present. Consequently, the DCA was conducted to showed that applying this prediction model has benefited patients when the threshold probability is in the range of 0.15–0.50 (Fig. 5).

**Table 3:** The features of 5 variables between training and validation cohorts

	Factors	Training cohort (*n* = 269)	Validation cohort (*n* = 108)	X^2^/W	*P*
Gender				0.69046	0.406
	Male	55	27		
	Female	214	81		
Multifocality				1.2256	0.268
	Negative	215	80		
	Positive	54	28		
Boundary				0.11573	0.734
	Clear	124	47		
	Unclear	145	61		
CLNM				2.4682	0.116
	Positive	78	41		
	Negative	191	67		
Age		43.50 ± 11.20	44.20 ± 11.40	14230	0.757
Tumor Size		0.63 ± 0.22	0.64 ± 0.22	13944	0.540

According to our study, CLNM may be related to several factors, including age, gender, tumor size, tumor multifocality, and ultrasound imaging-suggested tumor boundaries. The nomogram established in our study has moderate predictive ability for CLNM and can be applied to the clinical management of patients with PTMC.

## Discussion

Thanks to the development of high-resolution ultrasound, fine-needle aspiration biopsy (FNAB) as well as cytological and molecular diagnosis, more and more asymptomatic PTMC is being diagnosed at early stage [14]. Although the incidence of PTMC continues to rise, mortality rates have remained largely stable, with a 10-year survival rate of 94.6% [1, 15]. As a result, there is a growing debate about whether PTMC is over-diagnosed and over-treated. Some academics proposed that active surveillance (AS) was an effective management for PTMC, which meant surgery could be performed after the tumor progression was found during the monitoring process [16]. Based on active surveillance in low-risk PTMC, Ito et al. found that after 10 years of follow-up, only 8% of patients had a tumor size increase of more than 3 mm and only 3.8% had clinically confirmed lymph node metastases, and these patients still had a good prognosis with delayed surgery [2]. Thus, AS was suggested as an alternative option for the treatment of low-risk PTMC patients. A lot of academics from the United States, Italy and other countries have come to the same conclusion, believing that AS is a viable and safe alternative to immediate surgery for patients with cN0 PTMC [17–19]. Although many studies support this idea, there is still disagreement on the need to individualize patient care based on risk factors and patient preferences.

Currently, most academics believe that CLND is necessary for PTMC patients with cN1, but pCLND remains controversial for PTMC patients with cN0. On the one hand, ATA guidelines (version: 2015) and NCCN guidelines (version: 2022) suggest that pCLND is not recommended as a routine surgical procedure in cN0 PTMC patients without high risk factors [5, 9]. Some studies have shown that pCLND not only failed to improve the prognosis of patients, but also increased the risk of laryngeal return nerve injury and permanent hypoparathyroidism [5, 20]. On the other hand, some guidelines from different countries in Asia emphasized actively the significance of the routine pCLND for PTMC patients with cN0 [21]. It is helpful to reduce the rate of local recurrence and at the same time confirm the stage of thyroid cancer to provide guidance for subsequent treatments in terms of thyroid-stimulating hormone (TSH) suppression and radioactive iodine (RAI) therapy. PTMC patients were prone to develop CLNM at early stage [22]. In addition, it has been found that a second surgery in patients with post-operative recurrence is more difficult and has an increased probability of complications due to adhesions and altered natural anatomy [23]. Therefore, pCLND is significant to reduce the risk of reoperation for PTMC patients. Furthermore, ultrasound is the main way to evaluate the central lymph node involvement in PTMC patients preoperatively, but it has some limitations in clinical practice. The study conducted by YU et al. indicated that ultrasound has poor sensitivity in evaluating preoperative CLNM in patients with PTMC, ranging from 21.6%-38% [24]. Additionally, Kim and colleagues found that the false negative rate was 30% for preoperative ultrasound assessment of CLNM [25]. Consistent with this observation, in our study, all patients enrolled were cN0 PTMC patients, but 31.56% of them still had CLNM confirmed by the postoperative pathological results. While ultrasound has limitations, it remains a valuable non-invasive tool, and improving its accuracy through technological advancements or combined modalities could be an area for future research.

In current study, a total of 377 cN0 PTMC patients admitted to Thyroid and Breast Surgery of Second Affiliated Hospital of Fujian Medical University were enrolled to explore the risk factors associated with CLNM, resulting that gender, age, multifocality, tumor boundary and tumor size were the risk factors for CLNM in cN0 PTMC patients. Similarly, Pisanu A et al. identified gender, age, maximum tumor diameter, multifocality of the tumor, and vascular invasion as risk factors for the development of CLNM in patients with PTMC [26]. Additionally, the study conducted by YU X and colleagues indicated that male patients, age less than 45 years, multifocality of tumor, and tumor size larger than 5 mm in diameter were risk elements for CLNM [24]. In addition, Wei X et al. found that tumor location, aspect ratio and microcalcifications on ultrasound were associated with CLNM of PTMC patients [27]. The study by Gao Y et al. revealed that microcalcifications and poorly defined tumor boundaries were risk factors relevant to the development of CLNM in patients with PTMC [28]. In conclusion, some of the risk factors associated with CLNM in PTMC patients mentioned in other studies were not confirmed in this study such as tumor location, aspect ratio and microcalcifications, which may be related to the clinical experience of the ultra-sonographers and different ultrasound equipment.

The nomogram can make the prediction model simple and visualize the results in a graphical formal, which is widely used clinically in a variety of tumorigenesis and prognosis prediction, including breast cancer [29], gastric cancer [30], prostate cancer [31] and so on. However, most of the current nomograms of CLNM prediction in PTMC patients were established on the basis of postoperative pathological characteristics, and few models were proposed to make predictions about the CLNM preoperatively or intraoperatively [8, 32]. In current study, the nomogram was finally established based on clinical information, preoperative ultrasound examination and intraoperative frozen pathological characteristics, which is of great significance in the decision of surgical strategy for PTMC patients preoperatively. According to the nomogram risk model, the AUC value was 0.703 for the training set and 0.663 for the validation set, indicating that the model has moderate predictive efficacy. The calibration curves showed that the mean error between the actual probability of CLNM in PTMC patients and the predicted probability of this nomogram was less than 0.05, suggesting a good fit of the model. Ultimately, we evaluated the impact of the model on clinical decision making through clinical decision curves, meaning whether or not the patient would actually receive a clinical benefit. It was revealed that the model was able to contribute to improved clinical decisions and benefit patients within a certain threshold probability range (0.15–0.50). Although the nomogram shows promise, its effectiveness in clinical practice may need to be validated in a variety of settings and populations to ensure its robustness and generalizability. In summary, a prediction model for the development of CLNM in patients with cN0 PTMC was established based on clinical, ultrasound and intraoperative frozen pathological features. An aggressive pCLND was recommended to reduce the risk of tumor recurrence when the total score of a PTMC patients is higher than 120 based on the nomogram. Conversely, pCLND is not recommended when the total score of a PTMC patient is less than 120, which can reduce surgical complications. It is worth mentioning that for whether or not to perform pCLND, a balance needs to be struck between the benefits of pCLND and the possible reduction in quality of life due to surgical complications. In addition, the preference for AS and pCLND should be a shared decision between the patient and the surgeon. Therefore, adequate preoperative education and shared decision-making also play an important role in the management of pCLND.

However, there are still several limitations to this study. Firstly, as this study is a retrospective analysis, there is inevitably selective bias. Indeed, a number of other potential confounders, such as genetic mutations, environmental factors, and patients' lifestyles, which were not included in the study, may have influenced the results. Additionally, the pathological features and genotypes of FNA sample preoperatively should be further added into the future prediction model. Secondly, our model was only applied to cN0 PTMC, not for other subtypes of thyroid cancer. It is also important to incorporate the nomogram into a wider clinical framework, including endocrinologists, surgeons, pathologists, and even patient navigators, to ensure comprehensive care. Thirdly, this study was only conducted with single-center data from our medical center and lack of the external validation. Hence, subsequent evaluation with follow-up data from multicenter is indicated. Furthermore, it is suggested that future research may include prospective studies, the integration of genetic profiling, and the development of personalized medicine approaches for a more comprehensive investigation to guide clinical decision-making choices.

In conclusion, an effective nomogram for evaluating cervical central lymph node metastasis of cN0 PTMC was established based on the clinicopathological data from our medical center. Five variables were identified to be significantly related to CLNM, including age, gender, multifocality, tumor boundary and tumor size. For cN0 PTMC patients with a score higher than 120 according to the nomogram, clinicians can consider performing pCLND and making strict postoperative assessments.

### Supplementary Information

**Supplementary Material 1.**

## Data Availability

All data generated or analyzed during this study are included in this published article and its supplementary information files.
