# Supplementary material for: Development of a nomogram for prediction of central lymph node metastasis of papillary thyroid microcarcinoma
Source: BMC Cancer. 2024 Feb 20;24:235. doi: 10.1186/s12885-024-12004-3 (PMC10877775; doi:10.1186/s12885-024-12004-3)
Supplement: Supplementary file 1 — Supplementary Material 1. [file 12885_2024_12004_MOESM1_ESM.pdf]

Supplemental Table 1. Clinical information of 377 patients with PTMC

Patient Number	Gender	Age	Tumor Size	Multifocality	Position	Boundary	A/T	Microcalcification	Blood Supply	CLNM	Number of CLNM	Total Number of Lymph Nodes by CLND
1	Male	38	0.3	Negative	Middle	Unclear	>1	Negative	Negative	Positive	8	15
2	Male	45	0.9	Positive	Middle	Unclear	<=1	Negative	Negative	Positive	8	12
3	Male	50	1	Negative	Isthmus	Unclear	<=1	Positive	Positive	Positive	7	10
4	Female	50	0.7	Negative	Upper	Unclear	<=1	Positive	Positive	Positive	6	7
5	Male	57	1	Negative	Middle	Unclear	<=1	Positive	Positive	Positive	6	7
6	Female	37	1	Negative	Upper	Clear	<=1	Negative	Negative	Positive	5	7
7	Male	37	0.5	Negative	Middle	Unclear	>1	Positive	Negative	Positive	5	6
8	Female	33	1	Negative	Lower	Unclear	<=1	Positive	Positive	Positive	5	5
9	Female	34	0.3	Negative	Middle	Unclear	<=1	Positive	Negative	Positive	5	8
10	Female	40	0.8	Negative	Upper	Unclear	>1	Positive	Negative	Positive	5	7
11	Female	42	0.7	Negative	Lower	Unclear	>1	Negative	Negative	Positive	5	11
12	Female	50	0.8	Positive	Upper	Clear	<=1	Negative	Negative	Positive	4	8
13	Female	51	1	Negative	Middle	Clear	<=1	Negative	Positive	Positive	4	6
14	Male	42	0.5	Positive	Middle	Clear	<=1	Negative	Negative	Positive	4	4
15	Female	51	1	Negative	Middle	Clear	>1	Positive	Positive	Positive	4	6
16	Female	42	0.9	Negative	Upper	Clear	<=1	Positive	Negative	Positive	4	8
17	Female	61	0.6	Negative	Middle	Unclear	>1	Negative	Negative	Positive	4	8
18	Male	35	0.5	Negative	Middle	Unclear	>1	Negative	Negative	Positive	4	9
19	Male	36	0.9	Negative	Middle	Unclear	<=1	Positive	Positive	Positive	4	6
20	Female	39	0.9	Positive	Middle	Unclear	>1	Positive	Positive	Positive	4	5
21	Female	34	0.7	Negative	Lower	Unclear	>1	Positive	Negative	Positive	4	4
22	Female	49	0.8	Negative	Middle	Unclear	>1	Negative	Negative	Positive	4	7
23	Female	50	0.7	Positive	Middle	Unclear	<=1	Negative	Negative	Positive	4	7
24	Male	52	0.9	Negative	Lower	Unclear	<=1	Positive	Negative	Positive	4	6
25	Female	29	1	Negative	Upper	Clear	<=1	Positive	Positive	Positive	3	5
26	Male	37	0.8	Negative	Lower	Clear	<=1	Negative	Negative	Positive	3	3
27	Female	52	0.4	Negative	Upper	Clear	>1	Negative	Positive	Positive	3	4
28	Female	43	0.7	Positive	Lower	Clear	<=1	Positive	Negative	Positive	3	5
29	Female	62	0.7	Negative	Upper	Clear	>1	Positive	Negative	Positive	3	6
30	Female	69	0.9	Positive	Lower	Clear	>1	Positive	Negative	Positive	3	6
31	Female	53	0.8	Positive	Middle	Unclear	<=1	Negative	Positive	Positive	3	4
32	Male	32	0.3	Negative	Upper	Unclear	>1	Positive	Negative	Positive	3	3
33	Male	33	0.9	Positive	Middle	Unclear	<=1	Positive	Negative	Positive	3	4
34	Male	30	0.5	Negative	Lower	Unclear	<=1	Positive	Positive	Positive	3	3
35	Female	40	0.8	Positive	Upper	Unclear	<=1	Positive	Negative	Positive	3	12
36	Male	35	0.5	Positive	Lower	Unclear	<=1	Negative	Negative	Positive	3	3
37	Male	41	0.3	Positive	Middle	Unclear	<=1	Positive	Negative	Positive	3	5
38	Male	39	0.8	Negative	Middle	Unclear	>1	Positive	Negative	Positive	3	4
39	Female	73	0.8	Negative	Isthmus	Unclear	>1	Negative	Negative	Positive	3	8
40	Female	25	0.5	Negative	Upper	Unclear	<=1	Positive	Negative	Positive	3	12
41	Male	33	0.7	Negative	Lower	Clear	<=1	Positive	Negative	Positive	2	5
42	Female	36	0.7	Negative	Middle	Clear	>1	Positive	Negative	Positive	2	2
43	Female	47	0.6	Negative	Middle	Clear	>1	Negative	Negative	Positive	2	3
44	Female	56	0.9	Negative	Lower	Clear	>1	Positive	Negative	Positive	2	6
45	Female	30	0.6	Negative	Middle	Clear	<=1	Positive	Negative	Positive	2	6
46	Male	49	1	Negative	Middle	Clear	<=1	Positive	Negative	Positive	2	5
47	Female	62	0.8	Negative	Lower	Clear	<=1	Negative	Negative	Positive	2	2
48	Male	31	0.5	Negative	Lower	Clear	>1	Negative	Negative	Positive	2	8
49	Male	45	0.8	Negative	Middle	Clear	<=1	Positive	Negative	Positive	2	6
50	Female	63	0.2	Negative	Middle	Clear	>1	Positive	Negative	Positive	2	2
51	Female	24	0.4	Positive	Middle	Clear	<=1	Positive	Negative	Positive	2	13
52	Male	65	0.8	Positive	Middle	Unclear	<=1	Positive	Positive	Positive	2	4
53	Female	60	1	Positive	Middle	Unclear	<=1	Negative	Negative	Positive	2	10
54	Female	31	0.8	Negative	Upper	Unclear	<=1	Positive	Negative	Positive	2	11
55	Female	56	0.5	Negative	Middle	Unclear	<=1	Positive	Negative	Positive	2	9
56	Female	37	0.4	Negative	Middle	Unclear	<=1	Positive	Negative	Positive	2	6
57	Female	43	0.4	Positive	Upper	Unclear	<=1	Positive	Negative	Positive	2	13
58	Female	38	0.5	Negative	Middle	Unclear	<=1	Positive	Negative	Positive	2	6
59	Female	39	0.6	Positive	Middle	Unclear	>1	Positive	Negative	Positive	2	6
60	Female	30	0.8	Negative	Upper	Unclear	>1	Negative	Negative	Positive	2	6
61	Female	43	0.5	Positive	Middle	Unclear	>1	Positive	Positive	Positive	2	2
62	Male	32	0.5	Positive	Lower	Unclear	>1	Positive	Negative	Positive	2	12
63	Female	33	0.8	Positive	Middle	Unclear	<=1	Positive	Positive	Positive	2	7
64	Female	35	0.6	Positive	Middle	Unclear	<=1	Positive	Negative	Positive	2	6
65	Male	53	0.5	Negative	Upper	Unclear	>1	Positive	Positive	Positive	2	7
66	Female	65	1	Negative	Middle	Unclear	<=1	Positive	Positive	Positive	2	3
67	Female	34	0.7	Positive	Upper	Unclear	>1	Positive	Negative	Positive	2	15
68	Female	28	0.7	Negative	Middle	Unclear	<=1	Positive	Negative	Positive	2	2
69	Female	51	0.8	Negative	Lower	Unclear	<=1	Negative	Negative	Positive	2	7
70	Female	73	0.7	Positive	Upper	Unclear	>1	Negative	Negative	Positive	2	3
71	Female	47	0.8	Positive	Lower	Clear	>1	Negative	Positive	Positive	1	1
72	Female	30	0.5	Negative	Upper	Clear	<=1	Negative	Negative	Positive	1	5
73	Female	42	0.8	Positive	Middle	Clear	>1	Negative	Negative	Positive	1	4
74	Male	57	0.3	Negative	Middle	Clear	>1	Negative	Negative	Positive	1	5
75	Male	37	1	Negative	Lower	Clear	>1	Positive	Negative	Positive	1	3
76	Female	42	0.6	Negative	Upper	Clear	>1	Negative	Negative	Positive	1	15
77	Female	27	1	Positive	Middle	Clear	<=1	Positive	Positive	Positive	1	1
78	Female	43	0.6	Negative	Lower	Clear	>1	Negative	Positive	Positive	1	3
79	Female	55	0.4	Negative	Upper	Clear	>1	Negative	Negative	Positive	1	10
80	Female	49	0.6	Negative	Lower	Clear	>1	Negative	Negative	Positive	1	11
81	Male	35	0.5	Negative	Middle	Clear	<=1	Negative	Negative	Positive	1	2
82	Female	40	1	Positive	Lower	Clear	<=1	Positive	Negative	Positive	1	4
83	Female	37	0.5	Negative	Upper	Clear	<=1	Negative	Positive	Positive	1	1
84	Male	41	0.7	Positive	Lower	Clear	>1	Positive	Negative	Positive	1	5
85	Male	33	0.6	Negative	Lower	Clear	>1	Positive	Negative	Positive	1	2
86	Female	30	0.5	Positive	Lower	Clear	<=1	Positive	Negative	Positive	1	8
87	Female	46	0.7	Negative	Lower	Clear	>1	Negative	Negative	Positive	1	3
88	Male	47	0.8	Negative	Middle	Clear	<=1	Positive	Negative	Positive	1	5
89	Female	29	1	Negative	Upper	Clear	<=1	Negative	Negative	Positive	1	6
90	Female	39	0.9	Negative	Isthmus	Clear	<=1	Positive	Positive	Positive	1	4
91	Female	32	0.9	Negative	Middle	Clear	<=1	Positive	Negative	Positive	1	7
92	Female	38	0.6	Negative	Middle	Unclear	>1	Positive	Positive	Positive	1	4
93	Male	33	0.7	Negative	Middle	Unclear	>1	Negative	Positive	Positive	1	10
94	Female	30	0.8	Negative	Lower	Unclear	<=1	Positive	Negative	Positive	1	11
95	Female	34	0.8	Negative	Upper	Unclear	<=1	Negative	Negative	Positive	1	6
96	Female	30	1	Negative	Middle	Unclear	<=1	Negative	Positive	Positive	1	1
97	Male	37	0.5	Negative	Middle	Unclear	<=1	Positive	Negative	Positive	1	4
98	Female	37	0.6	Positive	Middle	Unclear	<=1	Positive	Negative	Positive	1	15
99	Female	33	0.7	Negative	Isthmus	Unclear	<=1	Positive	Negative	Positive	1	15
100	Female	36	0.6	Negative	Middle	Unclear	>1	Negative	Positive	Positive	1	1
101	Female	61	0.6	Positive	Middle	Unclear	>1	Negative	Positive	Positive	1	1
102	Female	38	0.3	Negative	Upper	Unclear	>1	Negative	Negative	Positive	1	4
103	Female	27	1	Negative	Lower	Unclear	<=1	Positive	Negative	Positive	1	2
104	Female	34	0.7	Negative	Upper	Unclear	<=1	Positive	Positive	Positive	1	5
105	Female	58	1	Negative	Middle	Unclear	>1	Positive	Negative	Positive	1	4
106	Male	31	0.9	Positive	Upper	Unclear	<=1	Positive	Negative	Positive	1	4
107	Female	46	0.8	Negative	Middle	Unclear	<=1	Negative	Negative	Positive	1	13
108	Female	57	0.9	Negative	Lower	Unclear	>1	Negative	Positive	Positive	1	2
109	Male	27	0.5	Negative	Middle	Unclear	>1	Positive	Negative	Positive	1	3

110	Female	41	1	Negative	Lower	Unclear	>1	Positive	Positive	Positive	1	6
111	Male	41	0.5	Negative	Middle	Unclear	>1	Positive	Negative	Positive	1	6
112	Female	47	0.3	Negative	Middle	Unclear	>1	Negative	Negative	Positive	1	6
113	Female	36	0.7	Negative	Middle	Unclear	>1	Negative	Negative	Positive	1	5
114	Female	60	0.6	Negative	Middle	Unclear	<=1	Negative	Negative	Positive	1	4
115	Female	37	0.4	Positive	Lower	Unclear	>1	Positive	Positive	Positive	1	2
116	Female	33	0.9	Positive	Middle	Unclear	<=1	Positive	Positive	Positive	1	6
117	Female	55	0.8	Negative	Upper	Unclear	>1	Positive	Negative	Positive	1	20
118	Male	24	0.6	Negative	Middle	Unclear	>1	Positive	Negative	Positive	1	8
119	Female	40	0.7	Negative	Lower	Unclear	>1	Positive	Negative	Positive	1	7
120	Female	36	0.5	Negative	Middle	Clear	>1	Negative	Negative	Negative	0	3
121	Female	55	0.8	Positive	Lower	Clear	<=1	Positive	Negative	Negative	0	5
122	Male	54	0.8	Negative	Middle	Clear	<=1	Negative	Positive	Negative	0	0
123	Female	54	0.7	Negative	Lower	Clear	<=1	Negative	Negative	Negative	0	3
124	Male	35	0.7	Negative	Middle	Clear	>1	Positive	Negative	Negative	0	5
125	Female	40	0.7	Positive	Middle	Clear	<=1	Positive	Negative	Negative	0	13
126	Female	38	0.5	Positive	Upper	Clear	>1	Positive	Negative	Negative	0	1
127	Female	37	0.8	Negative	Lower	Clear	<=1	Positive	Positive	Negative	0	1
128	Female	57	0.6	Negative	Upper	Clear	>1	Positive	Negative	Negative	0	2
129	Female	35	0.2	Negative	Upper	Clear	<1	Negative	Negative	Negative	0	1
130	Male	47	0.6	Positive	Lower	Clear	>1	Negative	Negative	Negative	0	2
131	Female	44	0.9	Negative	Middle	Clear	<=1	Positive	Positive	Negative	0	2
132	Female	43	0.6	Negative	Upper	Clear	>1	Positive	Negative	Negative	0	8
133	Female	52	0.5	Negative	Middle	Clear	>1	Positive	Negative	Negative	0	5
134	Male	42	0.7	Negative	Lower	Clear	<=1	Positive	Positive	Negative	0	2
135	Female	50	0.8	Negative	Middle	Clear	>1	Positive	Positive	Negative	0	1
136	Female	34	0.5	Positive	Middle	Clear	<=1	Positive	Positive	Negative	0	6
137	Male	42	0.9	Negative	Middle	Clear	<=1	Positive	Positive	Negative	0	1
138	Male	73	0.4	Negative	Middle	Clear	<=1	Positive	Negative	Negative	0	2
139	Male	50	0.8	Negative	Middle	Clear	<=1	Negative	Negative	Negative	0	3
140	Female	38	0.6	Negative	Isthmus	Clear	<=1	Positive	Negative	Negative	0	14
141	Male	38	0.6	Negative	Lower	Clear	>1	Negative	Negative	Negative	0	5
142	Female	40	0.7	Negative	Lower	Clear	<=1	Positive	Negative	Negative	0	4
143	Male	35	0.1	Negative	Middle	Clear	<=1	Negative	Positive	Negative	0	3
144	Female	47	0.9	Negative	Lower	Clear	<=1	Positive	Negative	Negative	0	3
145	Female	50	0.6	Negative	Middle	Clear	<=1	Negative	Negative	Negative	0	7
146	Male	57	0.6	Negative	Middle	Clear	<=1	Positive	Negative	Negative	0	2
147	Female	58	0.2	Negative	Lower	Clear	<=1	Negative	Negative	Negative	0	2
148	Female	47	0.5	Negative	Isthmus	Clear	<=1	Positive	Negative	Negative	0	5
149	Female	45	0.7	Negative	Lower	Clear	>1	Negative	Negative	Negative	0	13
150	Female	54	0.8	Positive	Middle	Clear	>1	Negative	Positive	Negative	0	5
151	Female	31	1	Negative	Lower	Clear	>1	Positive	Negative	Negative	0	3
152	Male	43	0.7	Negative	Middle	Clear	>1	Negative	Negative	Negative	0	2
153	Female	20	0.6	Negative	Middle	Clear	>1	Positive	Negative	Negative	0	3
154	Female	40	0.3	Negative	Lower	Clear	<=1	Negative	Negative	Negative	0	3
155	Female	43	0.9	Negative	Upper	Clear	<=1	Negative	Negative	Negative	0	9
156	Female	52	0.5	Positive	Lower	Clear	>1	Negative	Negative	Negative	0	10
157	Female	34	0.8	Negative	Lower	Clear	<=1	Positive	Negative	Negative	0	9
158	Male	43	0.4	Positive	Lower	Clear	>1	Positive	Negative	Negative	0	3
159	Female	62	0.5	Negative	Middle	Clear	<=1	Negative	Negative	Negative	0	13
160	Female	62	0.2	Negative	Upper	Clear	>1	Negative	Negative	Negative	0	2
161	Female	57	0.4	Negative	Upper	Clear	<=1	Positive	Negative	Negative	0	5
162	Female	58	0.6	Negative	Middle	Clear	>1	Negative	Negative	Negative	0	3
163	Male	24	0.6	Negative	Middle	Clear	<=1	Positive	Negative	Negative	0	7
164	Female	33	0.4	Negative	Lower	Clear	>1	Negative	Negative	Negative	0	2
165	Male	38	0.6	Negative	Lower	Clear	>1	Negative	Positive	Negative	0	3
166	Female	29	0.8	Negative	Middle	Clear	>1	Positive	Positive	Negative	0	3
167	Male	42	0.9	Negative	Upper	Clear	>1	Negative	Positive	Negative	0	1
168	Female	44	0.7	Negative	Middle	Clear	<=1	Positive	Positive	Negative	0	12
169	Male	51	0.2	Negative	Middle	Clear	<=1	Positive	Positive	Negative	0	11
170	Female	41	1	Negative	Middle	Clear	<=1	Negative	Positive	Negative	0	1
171	Female	34	0.3	Negative	Upper	Clear	>1	Negative	Negative	Negative	0	3
172	Male	56	0.6	Negative	Lower	Clear	<=1	Negative	Negative	Negative	0	6
173	Female	53	0.2	Negative	Lower	Clear	<=1	Positive	Positive	Negative	0	1
174	Male	44	1	Negative	Upper	Clear	>1	Positive	Positive	Negative	0	2
175	Female	41	0.8	Negative	Lower	Clear	<=1	Negative	Positive	Negative	0	4
176	Female	36	0.7	Positive	Lower	Clear	<=1	Negative	Negative	Negative	0	3
177	Female	51	0.5	Negative	Isthmus	Clear	>1	Negative	Positive	Negative	0	4
178	Female	47	0.6	Negative	Lower	Clear	<=1	Positive	Positive	Negative	0	9
179	Female	34	0.5	Negative	Upper	Clear	<=1	Positive	Positive	Negative	0	4
180	Female	66	0.9	Negative	Middle	Clear	<=1	Positive	Negative	Negative	0	3
181	Female	38	0.4	Negative	Lower	Clear	>1	Negative	Negative	Negative	0	5
182	Male	35	0.6	Negative	Middle	Clear	>1	Positive	Negative	Negative	0	1
183	Female	49	0.6	Negative	Lower	Clear	>1	Negative	Negative	Negative	0	5
184	Female	51	0.7	Negative	Lower	Clear	>1	Negative	Negative	Negative	0	6
185	Female	52	1	Negative	Middle	Clear	>1	Positive	Positive	Negative	0	4
186	Male	37	1	Negative	Lower	Clear	>1	Positive	Positive	Negative	0	6
187	Female	29	0.8	Negative	Lower	Clear	>1	Positive	Negative	Negative	0	3
188	Female	37	0.3	Positive	Lower	Clear	<=1	Negative	Negative	Negative	0	1
189	Female	29	0.7	Negative	Middle	Clear	<=1	Negative	Negative	Negative	0	2
190	Female	39	0.2	Negative	Middle	Clear	>1	Positive	Negative	Negative	0	2
191	Female	36	0.5	Positive	Middle	Clear	>1	Positive	Positive	Negative	0	16
192	Female	36	0.6	Positive	Lower	Clear	>1	Positive	Negative	Negative	0	7
193	Female	44	0.5	Negative	Lower	Clear	<=1	Positive	Negative	Negative	0	2
194	Female	48	0.9	Positive	Upper	Clear	<=1	Negative	Positive	Negative	0	6
195	Female	30	0.5	Positive	Middle	Clear	<=1	Positive	Positive	Negative	0	5
196	Male	56	0.5	Negative	Middle	Clear	>1	Negative	Negative	Negative	0	1
197	Female	50	0.5	Negative	Upper	Clear	<=1	Negative	Negative	Negative	0	2
198	Female	50	0.5	Positive	Middle	Clear	<=1	Positive	Negative	Negative	0	4
199	Female	47	0.7	Positive	Middle	Clear	<=1	Positive	Negative	Negative	0	6
200	Female	63	0.3	Negative	Lower	Clear	<=1	Negative	Positive	Negative	0	1
201	Male	47	0.5	Negative	Upper	Clear	>1	Negative	Negative	Negative	0	3
202	Female	29	0.8	Negative	Upper	Clear	<=1	Positive	Negative	Negative	0	4
203	Female	33	0.8	Negative	Upper	Clear	<=1	Positive	Positive	Negative	0	3
204	Female	36	0.3	Negative	Middle	Clear	<=1	Negative	Positive	Negative	0	5
205	Female	68	0.1	Negative	Lower	Clear	<=1	Negative	Positive	Negative	0	4
206	Male	37	0.8	Negative	Upper	Clear	<=1	Positive	Negative	Negative	0	2
207	Female	68	0.8	Negative	Lower	Clear	<=1	Negative	Negative	Negative	0	2
208	Female	50	0.2	Negative	Upper	Clear	>1	Negative	Positive	Negative	0	6
209	Female	49	0.8	Negative	Lower	Clear	>1	Negative	Positive	Negative	0	6
210	Male	33	0.6	Negative	Upper	Clear	<=1	Positive	Positive	Negative	0	12
211	Male	33	0.7	Negative	Lower	Clear	<=1	Positive	Positive	Negative	0	14
212	Female	31	0.5	Negative	Upper	Clear	<=1	Negative	Negative	Negative	0	2
213	Male	33	0.2	Negative	Lower	Clear	<=1	Negative	Negative	Negative	0	1
214	Female	40	0.4	Negative	Upper	Clear	>1	Negative	Negative	Negative	0	4
215	Female	44	0.5	Negative	Lower	Clear	<=1	Negative	Negative	Negative	0	3
216	Female	35	0.7	Negative	Middle	Clear	<=1	Negative	Positive	Negative	0	3
217	Female	39	0.4	Negative	Lower	Clear	>1	Positive	Positive	Negative	0	8
218	Female	61	0.9	Negative	Upper	Clear	>1	Negative	Positive	Negative	0	4
219	Female	37	0.8	Negative	Middle	Clear	<=1	Positive	Negative	Negative	0	1
220	Female	56	0.2	Negative	Lower	Clear	<=1	Negative	Negative	Negative	0	1

221	Female	48	0.8	Negative	Upper	Clear	<=1	Negative	Negative	Negative	0	5
222	Female	60	0.5	Positive	Lower	Clear	<=1	Negative	Negative	Negative	0	6
223	Female	32	0.2	Negative	Middle	Clear	>1	Negative	Positive	Negative	0	3
224	Female	76	0.8	Positive	Middle	Clear	>1	Positive	Positive	Negative	0	1
225	Female	63	0.2	Negative	Lower	Clear	<=1	Negative	Positive	Negative	0	4
226	Female	58	0.6	Negative	Upper	Clear	<=1	Negative	Positive	Negative	0	2
227	Male	41	1	Negative	Middle	Clear	<=1	Positive	Negative	Negative	0	3
228	Female	68	0.2	Negative	Upper	Clear	<=1	Positive	Positive	Negative	0	3
229	Female	37	0.6	Positive	Middle	Clear	>1	Negative	Positive	Negative	0	3
230	Female	57	0.3	Positive	Middle	Clear	<=1	Positive	Negative	Negative	0	3
231	Female	65	0.3	Negative	Middle	Clear	>1	Negative	Negative	Negative	0	7
232	Female	40	0.7	Positive	Upper	Clear	>1	Negative	Negative	Negative	0	7
233	Female	59	0.7	Negative	Upper	Clear	<=1	Positive	Positive	Negative	0	2
234	Female	35	0.6	Negative	Middle	Clear	>1	Negative	Negative	Negative	0	4
235	Female	31	0.5	Negative	Middle	Clear	>1	Positive	Negative	Negative	0	1
236	Female	46	0.6	Positive	Upper	Clear	<=1	Negative	Negative	Negative	0	1
237	Female	67	0.8	Negative	Middle	Clear	<=1	Negative	Negative	Negative	0	2
238	Female	37	0.8	Negative	Middle	Clear	>1	Positive	Negative	Negative	0	3
239	Female	37	0.5	Negative	Middle	Clear	>1	Negative	Negative	Negative	0	1
240	Female	61	1	Negative	Upper	Clear	<=1	Positive	Positive	Negative	0	3
241	Female	61	1	Negative	Lower	Clear	<=1	Positive	Positive	Negative	0	3
242	Female	57	0.8	Positive	Upper	Clear	>1	Positive	Negative	Negative	0	6
243	Female	59	0.2	Negative	Middle	Clear	<=1	Positive	Negative	Negative	0	1
244	Female	47	0.7	Negative	Middle	Clear	<=1	Positive	Negative	Negative	0	12
245	Female	32	0.9	Negative	Upper	Clear	>1	Positive	Negative	Negative	0	2
246	Female	47	0.8	Negative	Upper	Clear	>1	Negative	Negative	Negative	0	2
247	Female	32	0.6	Negative	Middle	Unclear	>1	Positive	Positive	Negative	0	11
248	Female	37	0.8	Negative	Lower	Unclear	<=1	Positive	Negative	Negative	0	6
249	Male	44	0.5	Negative	Middle	Unclear	>1	Positive	Positive	Negative	0	3
250	Female	44	0.7	Positive	Middle	Unclear	>1	Positive	Positive	Negative	0	5
251	Female	31	0.6	Positive	Middle	Unclear	>1	Positive	Negative	Negative	0	2
252	Female	43	0.6	Negative	Middle	Unclear	<=1	Negative	Positive	Negative	0	9
253	Male	40	0.7	Positive	Lower	Unclear	>1	Positive	Negative	Negative	0	4
254	Female	58	0.8	Negative	Middle	Unclear	>1	Positive	Positive	Negative	0	6
255	Female	33	0.8	Positive	Lower	Unclear	<=1	Positive	Negative	Negative	0	12
256	Female	68	0.7	Negative	Upper	Unclear	>1	Negative	Negative	Negative	0	3
257	Female	45	1	Negative	Middle	Unclear	<=1	Negative	Negative	Negative	0	4
258	Female	35	0.5	Negative	Middle	Unclear	>1	Positive	Negative	Negative	0	6
259	Male	47	0.5	Positive	Lower	Unclear	<=1	Positive	Negative	Negative	0	5
260	Female	30	0.2	Negative	Middle	Unclear	>1	Negative	Negative	Negative	0	9
261	Female	38	0.6	Negative	Lower	Unclear	<=1	Negative	Negative	Negative	0	8
262	Female	32	0.8	Negative	Upper	Unclear	<=1	Negative	Positive	Negative	0	7
263	Male	39	0.6	Positive	Lower	Unclear	>1	Positive	Negative	Negative	0	4
264	Female	33	0.8	Positive	Lower	Unclear	<=1	Negative	Negative	Negative	0	4
265	Female	32	0.7	Negative	Middle	Unclear	<=1	Negative	Negative	Negative	0	8
266	Female	49	0.9	Negative	Lower	Unclear	>1	Negative	Negative	Negative	0	2
267	Female	46	0.5	Negative	Lower	Unclear	>1	Negative	Negative	Negative	0	10
268	Female	45	0.7	Negative	Isthmus	Unclear	>1	Positive	Negative	Negative	0	4
269	Female	35	0.8	Negative	Middle	Unclear	>1	Positive	Negative	Negative	0	4
270	Female	52	1	Positive	Lower	Unclear	<=1	Positive	Negative	Negative	0	12
271	Female	21	0.4	Negative	Middle	Unclear	<=1	Negative	Negative	Negative	0	3
272	Female	53	0.7	Negative	Middle	Unclear	<=1	Negative	Positive	Negative	0	2
273	Male	33	0.6	Negative	Upper	Unclear	<=1	Negative	Negative	Negative	0	2
274	Female	46	0.2	Negative	Upper	Unclear	>1	Positive	Negative	Negative	0	2
275	Female	65	0.5	Negative	Middle	Unclear	>1	Negative	Positive	Negative	0	1
276	Female	37	0.9	Negative	Lower	Unclear	<=1	Positive	Positive	Negative	0	9
277	Male	42	0.4	Negative	Middle	Unclear	>1	Positive	Negative	Negative	0	10
278	Female	33	0.7	Negative	Upper	Unclear	>1	Positive	Negative	Negative	0	3
279	Female	67	0.4	Negative	Upper	Unclear	<=1	Positive	Positive	Negative	0	3
280	Female	26	0.2	Negative	Middle	Unclear	<=1	Positive	Positive	Negative	0	2
281	Female	33	0.6	Negative	Middle	Unclear	>1	Positive	Positive	Negative	0	16
282	Female	34	0.8	Positive	Lower	Unclear	<=1	Positive	Negative	Negative	0	6
283	Female	37	0.5	Negative	Upper	Unclear	>1	Negative	Positive	Negative	0	3
284	Female	34	0.5	Negative	Lower	Unclear	<=1	Negative	Negative	Negative	0	1
285	Female	24	1	Negative	Middle	Unclear	<=1	Positive	Negative	Negative	0	8
286	Female	44	0.5	Negative	Middle	Unclear	<=1	Positive	Negative	Negative	0	2
287	Female	54	0.6	Positive	Middle	Unclear	>1	Negative	Negative	Negative	0	3
288	Female	35	0.6	Negative	Middle	Unclear	>1	Negative	Negative	Negative	0	6
289	Female	48	0.8	Negative	Middle	Unclear	<=1	Positive	Negative	Negative	0	9
290	Female	49	0.8	Positive	Middle	Unclear	<=1	Positive	Negative	Negative	0	1
291	Female	42	0.5	Negative	Lower	Unclear	>1	Positive	Positive	Negative	0	2
292	Male	24	0.5	Negative	Middle	Unclear	<=1	Positive	Positive	Negative	0	2
293	Female	41	0.9	Negative	Middle	Unclear	<=1	Positive	Positive	Negative	0	4
294	Female	53	0.4	Negative	Middle	Unclear	<=1	Negative	Negative	Negative	0	4
295	Female	26	0.9	Negative	Isthmus	Unclear	<=1	Positive	Negative	Negative	0	8
296	Female	64	0.5	Negative	Upper	Unclear	<=1	Positive	Negative	Negative	0	9
297	Male	45	1	Negative	Middle	Unclear	<=1	Positive	Positive	Negative	0	4
298	Female	38	0.6	Positive	Lower	Unclear	<=1	Positive	Positive	Negative	0	1
299	Female	49	0.5	Negative	Middle	Unclear	>1	Positive	Positive	Negative	0	2
300	Female	68	0.5	Negative	Lower	Unclear	>1	Positive	Negative	Negative	0	6
301	Female	50	0.8	Positive	Upper	Unclear	>1	Positive	Positive	Negative	0	2
302	Male	28	0.7	Positive	Middle	Unclear	>1	Positive	Negative	Negative	0	3
303	Male	28	0.8	Positive	Middle	Unclear	>1	Positive	Negative	Negative	0	8
304	Female	55	0.3	Negative	Lower	Unclear	<=1	Negative	Negative	Negative	0	11
305	Female	51	0.4	Negative	Lower	Unclear	<=1	Negative	Negative	Negative	0	2
306	Female	51	0.3	Negative	Middle	Unclear	<=1	Negative	Negative	Negative	0	3
307	Female	35	0.4	Negative	Upper	Unclear	>1	Positive	Negative	Negative	0	4
308	Female	59	0.6	Negative	Middle	Unclear	>1	Positive	Negative	Negative	0	1
309	Female	44	0.8	Positive	Lower	Unclear	>1	Negative	Positive	Negative	0	3
310	Female	32	0.4	Negative	Middle	Unclear	>1	Positive	Positive	Negative	0	2
311	Female	41	0.8	Negative	Lower	Unclear	<=1	Positive	Negative	Negative	0	8
312	Female	43	0.3	Negative	Lower	Unclear	>1	Positive	Negative	Negative	0	1
313	Female	65	0.2	Negative	Lower	Unclear	<=1	Negative	Negative	Negative	0	2
314	Female	30	0.4	Negative	Middle	Unclear	>1	Positive	Negative	Negative	0	7
315	Female	43	0.8	Negative	Middle	Unclear	>1	Positive	Negative	Negative	0	9
316	Female	43	0.8	Negative	Middle	Unclear	>1	Negative	Negative	Negative	0	4
317	Female	35	0.4	Negative	Upper	Unclear	>1	Negative	Negative	Negative	0	3
318	Female	37	0.7	Negative	Lower	Unclear	<=1	Negative	Negative	Negative	0	3
319	Female	44	0.8	Negative	Upper	Unclear	<=1	Positive	Positive	Negative	0	6
320	Male	53	0.3	Positive	Lower	Unclear	>1	Negative	Negative	Negative	0	3
321	Female	37	0.6	Negative	Middle	Unclear	>1	Positive	Negative	Negative	0	5
322	Female	57	0.4	Negative	Middle	Unclear	>1	Positive	Negative	Negative	0	3
323	Female	53	0.8	Negative	Upper	Unclear	<=1	Positive	Negative	Negative	0	3
324	Female	46	0.6	Negative	Middle	Unclear	>1	Positive	Negative	Negative	0	4
325	Female	44	0.5	Negative	Middle	Unclear	<=1	Positive	Negative	Negative	0	4
326	Female	34	0.4	Negative	Middle	Unclear	>1	Negative	Positive	Negative	0	3
327	Male	57	0.8	Negative	Upper	Unclear	>1	Positive	Negative	Negative	0	1
328	Male	45	0.7	Negative	Lower	Unclear	<=1	Positive	Negative	Negative	0	2
329	Female	51	0.5	Negative	Middle	Unclear	>1	Negative	Negative	Negative	0	5
330	Female	50	0.6	Negative	Upper	Unclear	<=1	Positive	Negative	Negative	0	7
331	Female	34	0.7	Negative	Middle	Unclear	<=1	Negative	Negative	Negative	0	3

332	Female	31	0.4	Negative	Upper	Unclear	<=1	Positive	Positive	Negative	0	4
333	Female	57	1	Negative	Middle	Unclear	<=1	Positive	Positive	Negative	0	7
334	Female	36	0.6	Negative	Upper	Unclear	<=1	Positive	Negative	Negative	0	5
335	Female	36	0.5	Negative	Lower	Unclear	>1	Positive	Negative	Negative	0	3
336	Female	33	0.7	Negative	Middle	Unclear	>1	Negative	Negative	Negative	0	6
337	Male	55	0.5	Negative	Middle	Unclear	>1	Negative	Negative	Negative	0	4
338	Female	49	0.6	Negative	Middle	Unclear	>1	Positive	Negative	Negative	0	6
339	Female	67	0.4	Negative	Lower	Unclear	<=1	Negative	Negative	Negative	0	2
340	Male	29	0.7	Negative	Middle	Unclear	>1	Positive	Positive	Negative	0	6
341	Female	29	0.7	Negative	Upper	Unclear	>1	Negative	Negative	Negative	0	6
342	Male	36	0.8	Negative	Lower	Unclear	<=1	Positive	Positive	Negative	0	4
343	Female	41	0.5	Positive	Lower	Unclear	>1	Negative	Negative	Negative	0	1
344	Female	27	0.2	Negative	Middle	Unclear	>1	Negative	Negative	Negative	0	1
345	Female	46	0.3	Positive	Lower	Unclear	>1	Positive	Positive	Negative	0	3
346	Female	47	0.7	Negative	Middle	Unclear	>1	Negative	Positive	Negative	0	3
347	Female	33	0.5	Negative	Middle	Unclear	<=1	Positive	Positive	Negative	0	3
348	Female	39	0.9	Negative	Upper	Unclear	<=1	Positive	Negative	Negative	0	6
349	Female	34	0.8	Negative	Middle	Unclear	<=1	Positive	Negative	Negative	0	9
350	Female	35	0.6	Positive	Upper	Unclear	<=1	Negative	Positive	Negative	0	2
351	Female	52	0.7	Negative	Lower	Unclear	>1	Positive	Negative	Negative	0	4
352	Female	53	0.1	Negative	Middle	Unclear	>1	Positive	Negative	Negative	0	2
353	Female	35	0.5	Negative	Lower	Unclear	>1	Negative	Negative	Negative	0	5
354	Female	58	0.5	Positive	Lower	Unclear	<=1	Positive	Positive	Negative	0	4
355	Male	71	0.5	Negative	Upper	Unclear	<=1	Positive	Negative	Negative	0	1
356	Female	54	0.9	Negative	Upper	Unclear	>1	Negative	Negative	Negative	0	2
357	Female	48	0.4	Negative	Middle	Unclear	>1	Negative	Negative	Negative	0	10
358	Female	55	0.5	Negative	Lower	Unclear	<=1	Positive	Negative	Negative	0	8
359	Female	33	0.6	Negative	Middle	Unclear	>1	Positive	Negative	Negative	0	5
360	Female	36	0.8	Negative	Lower	Unclear	>1	Positive	Negative	Negative	0	6
361	Female	46	0.3	Positive	Upper	Unclear	>1	Negative	Negative	Negative	0	3
362	Female	42	0.5	Negative	Lower	Unclear	<=1	Negative	Negative	Negative	0	2
363	Female	53	0.7	Negative	Upper	Unclear	<=1	Positive	Negative	Negative	0	3
364	Female	56	0.7	Negative	Upper	Unclear	>1	Positive	Negative	Negative	0	3
365	Male	28	0.3	Negative	Middle	Unclear	>1	Positive	Negative	Negative	0	4
366	Female	63	0.7	Negative	Middle	Unclear	<=1	Positive	Negative	Negative	0	4
367	Female	54	0.3	Negative	Middle	Unclear	>1	Negative	Negative	Negative	0	4
368	Female	47	0.5	Negative	Middle	Unclear	>1	Negative	Negative	Negative	0	4
369	Female	42	0.3	Negative	Upper	Unclear	>1	Negative	Positive	Negative	0	5
370	Female	39	0.9	Positive	Upper	Unclear	>1	Positive	Negative	Negative	0	14
371	Female	53	0.3	Positive	Middle	Unclear	>1	Negative	Negative	Negative	0	4
372	Female	55	0.5	Negative	Upper	Unclear	<=1	Positive	Positive	Negative	0	5
373	Female	60	0.8	Negative	Middle	Unclear	<=1	Negative	Positive	Negative	0	3
374	Male	43	0.8	Positive	Middle	Unclear	>1	Negative	Negative	Negative	0	4
375	Male	26	0.7	Negative	Lower	Unclear	>1	Negative	Negative	Negative	0	3
376	Female	45	0.4	Negative	Lower	Unclear	>1	Positive	Positive	Negative	0	4
377	Female	49	0.5	Negative	Middle	Unclear	<=1	Negative	Positive	Negative	0	2
